# A toxicological assessment of *Ganoderma lucidum* and *Cordyceps militaris* mushroom powders

**DOI:** 10.3389/ftox.2024.1469348

**Published:** 2024-10-30

**Authors:** Paola P. Chrysostomou, Elaine Freeman, Mary M. Murphy, Ankit Chaudhary, Nazia Siddiqui, Julie Daoust

**Affiliations:** ^1^ Exponent Inc., Center for Chemical Regulation and Food Safety, Washington, DC, United States; ^2^ Department of Pharmaceutical Technology, Meerut Institute of Engineering and Technology (MIET), Meerut, India; ^3^ Scientific Affairs, M2 Ingredients, Vista, CA, United States

**Keywords:** Reishi, Ganoderma lucidum, Cordyceps militaris, mushroom, mycelium, fruiting body, toxicity

## Abstract

*Gonoderma lucidum (G. lucidum)* and *Cordyceps militaris (C. militaris)* are among the many mushrooms known for their long history of use in traditional medicine. Wildcrafted sources of mushrooms including *G. lucidum* and *C. militaris* can be limited from a scarcity and quality perspective, but solid fermentation processes in cultivation settings can provide an efficient way to deliver whole mushroom preparations of a consistent composition. Despite the historical use of these mushrooms, few published reports have explored their potential subchronic oral toxicity or genotoxicity, either from specific components or whole mushroom preparations. The purpose of this study was to assess the potential for acute toxicity, subchronic toxicity, and genotoxicity of powders produced from *G. lucidum* mycelial biomass and fruiting body (“Organic Reishi M2-102-02 powder”) cultured on oats, and *C. militaris* mycelial biomass, stroma, and fruiting body (“Organic Cordyceps M2-116-04 powder”) cultured on oats. Results of the testing demonstrate that both Organic Reishi M2-102-02 powder and Organic Cordyceps M2-116-04 powder were not acutely toxic, did not exhibit subchronic oral toxicity in rats at doses up to the highest dose tested of 2,000 mg/kg bw/day, and did not have genotoxic potential based on *in vitro* and *in vivo* genotoxicity assays.

## 1 Introduction

Mushrooms from the genus *Ganoderma* and *Cordyceps* are among the many mushrooms known for their long history of use in traditional medicines*. Ganoderma lucidum* (*G. lucidum;* commonly known as Reishi) is a wood decaying basidiomycete typically found on hardwood trees. *G. lucidum* has a sporocarp that is shelf-like and presents a stipe (Wachtel-Galor et al., 2011; Sułkowska-Ziaja et al., 2023; Kumari et al., 2024). *Cordyceps militaris* (*C. militaris*) is an ascomycete characterized by club-shaped orange or scarlet stromae and fruiting bodies and commonly grows on lepidopteran larvae and pupae (Jhou et al., 2018).

*G. lucidum* has been associated with antioxidant, antitumor, anti-inflammatory, anti-diabetic, hypolipidemic, and immunomodulatory effects (Sułkowska-Ziaja et al., 2023; Bishop et al., 2015; Lu et al., 2020). The principal components of *G. lucidum* include polysaccharides, glycoproteins, and triterpenoids including ganoderic acids (Ekiz et al., 2023; Lu et al., 2020). *C. militaris* has been associated with ergogenic, antitumor, antioxidant, and immunomodulatory effects (Jędrejko et al., 2021). The principal components of *C. militaris* also include various carotenoids, cordycepin, D-mannitol (cordycepic acid), γ-aminobutyric acid, N^6^-(2-hydroxyethyl)-adenosine, and various polysaccharides (Jędrejko et al., 2021; Wu et al., 2021).

*G. lucidum* is a woody mushroom and is considered inedible as a culinary mushroom whereas *C. militaris* is sometimes used in Asian cuisine. Preparations of these mushrooms are typically available for medicinal or functional purposes as dried and powdered preparations of fruiting bodies or mycelia, or extracts of all or some fungal parts. Wildcrafted sources of both *G. lucidum* and *C. militaris,* however, are limited, thus preparations from liquid fermentation or solid-state fermentation processes provide a means to highly efficient, consistent, year-round production and allow for better quality control of the final product (Wachtel-Galor et al., 2011; Jhou et al., 2018).

Although both *G. lucidum* and *C. militaris* have a long history of use as traditional medicines, rather limited information on their potential toxicity has been reported in the published literature (Ahmad et al., 2021; Jędrejko et al., 2021). Toxicity testing to support the safety of powders produced from *G. lucidum* mycelial biomass and fruiting body (“Organic Reishi M2-102-02 powder”) and C. militaris mycelial biomass, stroma, and fruiting body (“Organic Cordyceps M2-116-04 powder”) cultured on organic whole oats was conducted. The potential for acute toxicity, subchronic toxicity, and genotoxicity of Organic Reishi M2-102-02 powder and Organic Cordyceps M2-116-04 powder were evaluated.

## 2 Materials and methods

Organic Reishi M2-102-02 powder and Organic Cordyceps M2-116-04 powder were evaluated for the potential to cause acute toxicity, subchronic toxicity, and genotoxicity. The two materials were evaluated in separate toxicology studies conducted at the Meerut Institute of Engineering & Technology (MIET) following identical protocols. The conditions conform to MIET’s standard operating system that are based on the institute’s internal “Guide for the Care and Use of Experimental Animals.” All studies were reported to be conducted under Good Laboratory Practice (GLP).

### 2.1 Test material

M2 Ingredients Inc. provided the test materials, Organic Reishi M2-102-02 and Organic Cordyceps M2-116-04. Organic Reishi M2-102-02 powder consists of *G. lucidum* mycelial biomass and fruiting body cultured on whole certified organic oats (*Avena sativa),* dehydrated gently to break down the chitinous cell walls of the fungi and then milled into a powder. Organic Cordyceps M2-116-04 powder consists of *C. militaris* mycelial biomass, stroma, and fruiting body cultured on organic whole oats, dehydrated and milled into a powder. Identities were confirmed with species positive identification specifications using DNA sequencing of master tissue culture, taxonomic and visual monitoring of morphology, and growth metrics during the growing cycle. The concentration of beta-glucan in the Organic Reishi M2-102-02 powder and Organic Cordyceps M2-116-04 powder test materials was 29.1% w/w and 37.2% w/w, respectively. Organic Cordyceps M2-116-04 powder was also characterized by the presence of cordycepin (0.468 mg/g). Other characteristics of the test materials are summarized in Table 1.

**TABLE 1:** Analytical characterization of Organic Reishi M2-102-02 powder and Organic Cordyceps M2-116-04 powder used in the toxicity studie**s**.

	Test material	
Characteristic	Organic Reishi M2-102-02 powder	Organic Cordyceps M2-116-04 powder
Lot number	L22080102A	L22080804A
Appearance	Brown powder	Light brown powder
Beta glucan content (% w/w)	29.1	37.2
Cordycepin (mg/g)	—	0.468
Particle size (through 60 mesh)	≥95%	≥95%
Moisture (% w/w)	3.9	3.5

### 2.2 Acute oral toxicity

The acute oral toxicity study designs were based on the Organisation for Economic Cooperation and Development (OECD) Guidelines for Testing of Chemicals, No. 425 (OECD, 2022). In each test, five female Wistar rats were administered Organic Reishi M2-102-02 powder or Organic Cordyceps M2-116-04 powder in purified water by single oral gavage dose at 2000 mg/kg bw and observed for up to 14 days. Additionally, groups of three female rats/dose were administered Organic Reishi M2-102-02 powder or Organic Cordyceps M2-116-04 powder in purified water by single oral gavage dose at 61, 195, or 625 mg/kg bw and observed up to 14 days. All animals acclimated to the laboratory for 5 days prior to dosing. General clinical observations for muscle activity (locomotion, muscle coordination, catatonia, tremor, and convulsion), reflex activity (visual place response, writhing response, tail pinch response, and piloerection), and secretory activity (lacrimation, salivation, sniffing, and defecation) were made, and respiratory and heart rates were examined. Evaluations of motor activity and grip strength were reported. Body weight was measured on Day 0 prior to administration of test material and on Days 1, 7, and 14. Food consumption per animal cage was reported daily. At the end of the observation period, the surviving animals were sacrificed using pentobarbital and were subjected to gross necropsy, including gross pathological evaluation.

### 2.3 90-Day oral toxicity

The 90-day oral toxicity studies were based on OECD Guidelines for Testing of Chemicals, No. 408 (OECD, 2018). In each study, Wistar rats (10/sex/group) were administered test material suspended in purified water by oral gavage at doses of 0, 500, 1,000, or 2000 mg/kg bw/day for 90 days. Animals were fasted overnight prior to administration and 2–4 h after administration. Water was provided *ad libitum* and feed was provided daily*.* All animals acclimated to the laboratory for 5 days prior to dosing. Environmental conditions included 10–15 air changes per hour, 22°C ± 3°C, relative humidity 40%–60% and illumination cycle set to 12 h artificial fluorescent light and 12 h dark. All animals were observed daily for morbidity, mortality, and general clinical observations (muscle activity including locomotion, muscle coordination, catatonia, tremor, and convulsive episode, reflex activity including visual place response, writhing response, tail pinch response, and piloerection, secretory activity including lacrimation, salivation, sniffing, and defecation, respiratory, and heart rate). Body weight was measured on Day 0 prior to test item administration and every 12th day thereafter.

Clinical pathology evaluations were performed on all animals at the end of the study, including evaluations of hematology (hemoglobin, red blood cell count, white blood cell count, platelet count, packed cell volume, mean corpuscular hemoglobin concentration (MCHC), mean corpuscular hemoglobin, mean corpuscular volume (MCV), differential leukocyte count including neutrophils, eosinophils, lymphocytes, and monocytes), clinical chemistry (total protein, total cholesterol, triglycerides, albumin, creatinine, alanine aminotransferase, aspartate aminotransferase (AST), alkaline phosphatase, blood urea nitrogen, bilirubin total and direct, glucose, uric acid, thyroid hormones), and coagulation (clotting time measurement). Urinalysis (color, clarity, pH, protein, glucose, bilirubin, and microscopic examination) was performed on all animals in the last week before termination (Day 90). Food and water were not provided to the animals during the urine and blood collection period.

Necropsy was performed on all animals and absolute weights of adrenals, brain, kidneys, lungs, heart, spleen, testes, uterus, and ovaries were recorded. Organ weights relative to body weights were calculated based on the absolute weight and the body weight of the animal. Histopathological evaluations were conducted on control and high dose group animals and included liver, kidneys, lungs, heart, brain, stomach, spleen, testes (including seminiferous tubules), uterus (including cervix), and ovaries. The lungs of animals in the low- and mid-dose groups were subject to histopathological examination for evidence of infection.

### 2.4 Bacterial reverse mutation assay

The bacterial reverse mutation assay study design was based on the OECD Guidelines for Testing of Chemicals, No. 471 (Organisation for Economic and Cooperative Development, 2002) using *Salmonella typhimurium* strains TA98, TA100, TA1535, and TA, and *Escherichia coli* strain WP2 uvrA in the presence and absence of exogenous metabolic activation system (S9 fraction liver extract). The strains were purchased from Environmental Bio-Detection Products Inc. (Mississauga Ontario, Canada). The metabolic activation system, Aroclor™ 1254-induced rat liver S9 (frozen dried), was purchased from Xenotech LLC (USA). The testing procedure consisted of a cytotoxicity test, a definitive assay, and a confirmatory repeat assay up to 5,000 µg/plate of each test substance. The metabolic activation system used in the assays was co-factor supplemented Arcolor™ 1254-induced rat liver S9 at 5% (definitive assay) and 10% (repeat assay) concentrations. Dimethyl sulfoxide was used as the vehicle for the test powders. Plates were prepared in triplicate and revertant colonies were counted using a digital colony counter. The mean and standard deviation were calculated for each set of triplicate plates.

### 2.5 *In Vivo* micronucleus test

2.6 Organic Reishi M2-102-02 powder and Organic Cordyceps M2-116-04 powder were tested *in vivo* for the potential to cause clastogenicity and/or aneugenicity. The test design was based on OECD 474 (OECD, 2014). The test items were prepared in purified water, phosphate buffered saline was used as vehicle control, and cyclophosphamide (50 mg/kg bw) was used as the positive control. The positive control material was supplied by Goel Scientific Pvt. Ltd. (manufactured by Sigma, USA), and the mice were provided by Lala Lajpat Rai University. In each study, Swiss albino mice (5/sex/group) were administered 0, 300, 1,000, 2000, or 2000 (satellite group) mg/kg bw of the Organic Reishi M2-102-02 powder or Organic Cordyceps M2-116-04 powder or positive control by oral gavage. Each test item was administered to each group in two doses at 14-h intervals. The positive control, negative control, and treatment groups were sacrificed after 24 h and the satellite group was sacrificed after 48 h. Bone marrow smears were collected, fixed, stained, and examined under light microscopy at ×100 magnification. The incidence of micronucleated cells per 2000 polychromatic erythrocytes (PCE)-blue stained immature cells per animal was scored. The number of normochromatic erythrocytes pink stained mature cells associated with 1,000 erythrocytes was counted and scored for incidence of micronuclei. The ratio of polychromatic to normochromatic erythrocytes was calculated together with appropriate group mean values and standard deviations.

### 2.6 Statistical analysis

Statistical analysis for the 90-day oral toxicity study was conducted using R and Excel. Means and standard deviations were calculated for males and females by dose group for each safety parameter evaluated (hematology, clinical chemistry, body weight, and organ weight). Two-way ANOVA was conducted to calculate statistical significance (*p* > 0.05) between groups by sex. Statistical analysis for the *in vivo* micronucleus test was conducted using one-way ANOVA.

## 3 Results

### 3.1 Acute oral toxicity

In acute oral toxicity studies in rats, no mortality was observed up to 14 days after administration of up to 2000 mg/kg bw Organic Reishi M2-102-02 powder or 2000 mg/kg bw Organic Cordyceps M2-116-04 powder. No clinical signs, body weight changes, or macroscopic findings were observed. Based on the results of these studies, Organic Reishi M2-102-02 powder and Organic Cordyceps M2-116-04 powder were not lethal up to the limit dose of 2000 mg/kg bw, thus LD_50_ values were >2000 mg/kg bw for each test substance. The summary results from the acute oral toxicity studies with Organic Cordyceps M2-116-04 and Organic Resihi M2-102-02 powders are provided in (Supplementary Tables 1, 2).

### 3.2 90-Day oral toxicity

#### 3.2.1 Organic Reishi M2-102-02 powder

**FIGURE 1:**
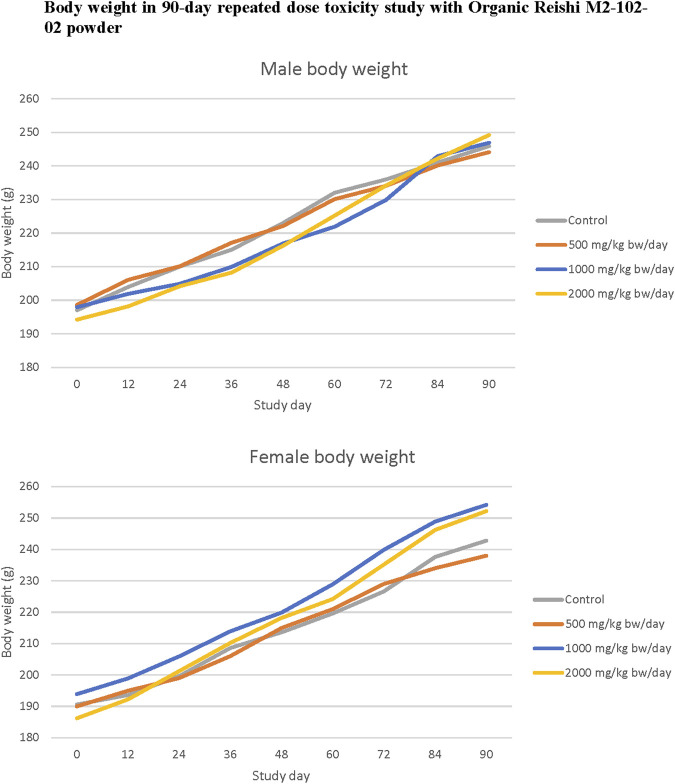
Body weight in 90-day repeated dose toxicity study with Organic Reishi M2-102-02 powder.

No statistically significant or treatment related effects mortality, clinical signs, ophthalmic abnormalities, urinalysis finding, or macroscopic or microscopic findings were observed in male or female rats in any dose group administered Organic Reishi M2-102-02 powder in the 90-day oral toxicity study, and no statistically significant changes in body weight were observed (Figure 1). Absolute spleen weights of high dose females were significantly increased compared with controls (*p* < 0.05). Ovary weights (absolute and relative to body weight) of high dose females and testes weights (absolute and relative to body weight) of high dose males were significantly increased compared with controls (*p* < 0.05). No microscopic correlates were observed in histopathological evaluation of the spleen, testes, or ovaries of high dose rats. No statistically significant changes in hematological parameters were reported for male rats at any dose group when compared to control. Total white blood cell count was significantly decreased in females at all doses compared to controls (*p* < 0.05), while MCV and MCHC were significantly increased in mid and high dose group females compared to controls (*p* < 0.05). No statistically significant changes in clinical chemistry parameters were reported for female rats at any dose group when compared to control. AST was significantly increased in high dose males compared to controls (*p* < 0.05). All changes did not show a dose-response relationship or were within normal variability and were not considered adverse. Therefore, the no observed adverse effect level (NOAEL) for Organic Reishi M2-102-02 powder was 2000 mg/kg bw/day based on no adverse effects reported at the highest dose level tested. A summary of the results of the 90-day oral toxicity study with Organic Reishi M2-102-02 powder is shown in Table 2. The complete organ weight, hematological, and clinical chemistry parameters are provided in (Supplementary Tables 3–5).

**TABLE 2:** Statistically significant parameters in the 90-day repeated dose study with Organic Reishi M2-102-02 powder.

	Males				Females			
Dose group (mg/kg bw/day)	Control	500	1,000	2000	Control	500	1,000	2000
Absolute organ weight								
Spleen (g)	0.8 ± 0.0	0.8 ± 0.0	0.8 ± 0.0	0.8 ± 0.0	0.8 ± 0.0	0.8 ± 0.0	0.8 ± 0.0	0.9 ± 0.0*
Testes (g)	2.3 ± 0.0	2.5 ± 0.0	2.4 ± 0.1	2.6 ± 0.0	-	-	-	-
Ovary (mg)	—	—	—	—	64.7 ± 1.1	65.7 ± 3.8	65.0 ± 4.8	74.0 ± 2.9*
Relative organ weight to body weight (%)								
Ovary	—	—	—	—	0.027 ± 0.000	0.028 ± 0.000	0.026 ± 0.000	0.029 ± 0.000*
Testes	0.95 ± 0.00	1.01 ± 0.00*	0.96 ± 0.00	1.04 ± 0.00*	—	—	—	—
Hematology								
Total WBC count (x10^3^/µL)	6.9 ± 1.2	5.0 ± 1.2*	6.5 ± 0.9	6.8 ± 0.7	9.5 ± 1.1	7.4 ± 1.2*	7.8 ± 0.9*	7.7 ± 1.0*
PCV (%)	38.2 ± 0.8	43.3 ± 1.1*	39.4 ± 3.8	35.9 ± 3.0	42.0 ± 1.5	45.0 ± 1.9	43.1 ± 2.4	43.4 ± 3.8
Platelet count (lakh/cmm)	670.6 ± 63.7	723.8 ± 39.9	848.2 ± 87.9*	690.7 ± 88.6	915.3 ± 111.9	815.2 ± 67.2	866.9 ± 82.4	761.5 ± 127.4*
MCV (fL)	41.6 ± 1.5	51.6 ± 2.6*	59.2 ± 1.1*	56.2 ± 2.2*	48.0 ± 2.4	51.8 ± 1.6	60.3 ± 1.1*	59.7 ± 2.7*
MCHC (g/dL)	28.6 ± 0.7	27.9 ± 0.4	18.2 ± 1.6*	31.0 ± 1.0*	29.0 ± 1.5	27.8 ± 0.5	33.5 ± 2.6*	32.3 ± 1.2*
Lymphocytes (%)	62.6 ± 1.3	67.1 ± 2.5*	66.4 ± 4.0*	62.8 ± 2.5	70.1 ± 2.9	68.5 ± 2.3	70.4 ± 2.0	72.6 ± 2.2
Neutrophils (%)	25.0 ± 2.1	28.7 ± 1.3*	26.6 ± 3.0	26.0 ± 2.2	29.8 ± 2.8	27.7 ± 1.2	31.6 ± 3.6	32.6 ± 2.9
Clinical chemistry								
AST (IU/L)	57.6 ± 7.0	65.6 ± 6.8	61.2 ± 2.6	69.7 ± 1.6*	67.4 ± 8.5	65.0 ± 10.1	57.7 ± 6.3	69.8 ± 10.1
ALT (IU/L)	22.7 ± 5.3	26.9 ± 4.5	25.8 ± 13.8	33.2 ± 7.5	22.9 ± 6.9	26.9 ± 4.5	28.5 ± 5.9	30.2 ± 4.6
BUN (mg/dL)	21.1 ± 2.8	22.3 ± 3.3	26.9 ± 0.5*	20.2 ± 2.5	21.7 ± 2.1	24.0 ± 1.1	28.2 ± 1.8*	21.4 ± 2.0

MCH, Mean corpuscular hemoglobin; MCHC, Mean corpuscular hemoglobin concentration; MCV, Mean corpuscular volume; PCV, Packed cell volume; Total RBC, Total red blood cell; Total WBC, Total white blood cell; Alanine Aminotransferase - ALT; Aspartate aminotransferase - AST; Blood urea nitrogen - BUN.

Data shown as mean ± SD.

*Statistical significance compared to control, *p* < 0.05.

N = 10 animals/sex/group.

Note: Some values appear to be zero (0.0, 0.00, or 0.000) due to rounding.

#### 3.2.2 Organic Cordyceps M2-116-04 powder

**FIGURE 2:**
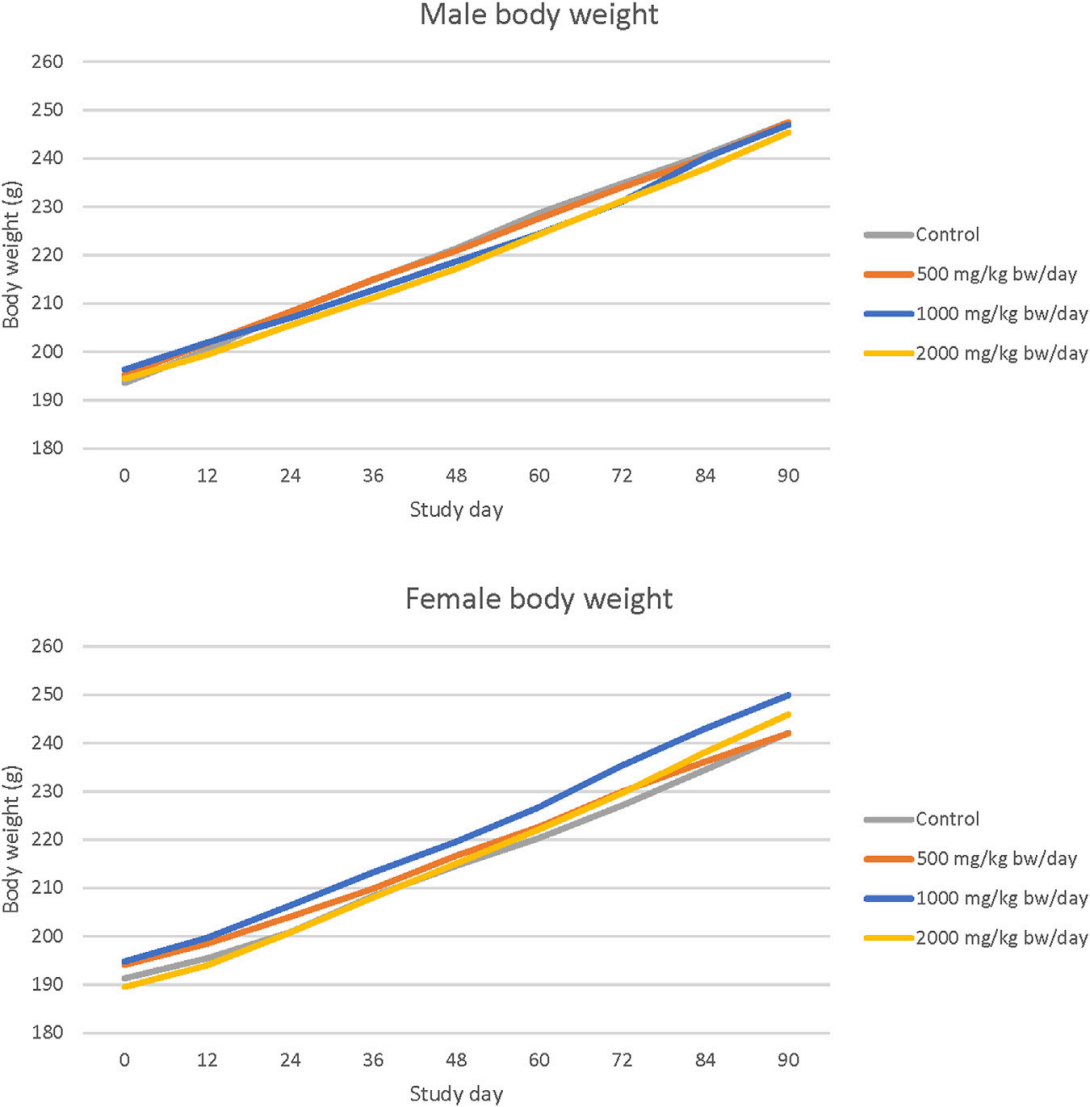
Body weight in 90-day repeated dose toxicity study with Organic Cordyceps M2-116-04 powder.

No statistically significant or treatment related effects on mortality, clinical signs, ophthalmic abnormalities, changes in hematology or clinical chemistry parameters, urinalysis findings, or macroscopic or microscopic findings were observed in male or female rats in any dose group in the 90-day study with Organic Cordyceps M2-116-04 powder. No statistically significant changes in body weight were observed (Figure 2). Ovary weights (absolute and relative to body weight) of all dosed females and testes weights (absolute and relative to body weight) of high dose males were significantly increased compared with controls (*p* < 0.05). Brain weight relative to body weight was significantly increased compared to controls in high dose females (*p* < 0.05), with no histopathological correlates. No microscopic correlates were observed in histopathological evaluation of the testes or ovaries of high dose rats. All changes did not show a dose-response relationship or were within normal variability and were not considered adverse. The NOAEL for Organic Cordyceps M2-116-04 powder was 2000 mg/kg bw/day based on no adverse effects reported at the highest dose level tested. A summary of the results of the 90-day oral toxicity study with Organic Cordyceps M2-116-04 powder is shown in Table 3. The complete organ weight, hematological, and clinical chemistry parameters are provided in (Supplementary Tables 6–8).

**TABLE 3:** Statistically significant parameters in the 90-day repeated dose study with Organic Cordyceps M2-116-04 powder.

	Males				Females			
Dose group (mg/kg bw/day)	Control	500	1,000	2000	Control	500	1,000	2000
Absolute organ weight								
Brain (g)	2.1 ± 0.1	2.1 ± 0.1	2.1 ± 0.0	2.2 ± 0.1	2.1 ± 0.0	2.0 ± 0.0	2.1 ± 0.0	2.4 ± 0.1*
Testes (g)	2.6 ± 0.0	2.5 ± 0.0*	2.3 ± 0.1	2.5 ± 0.1*	—	—	—	—
Ovary (mg)	—	—	—	—	66.0 ± 4.9	61.0 ± 3.0*	59.9 ± 0.0*	69.9 ± 0.0*
Relative organ weight to body weight								
Brain (%)	0.9 ± 0.0	0.8 ± 0.0	0.8 ± 0.0	0.9 ± 0.0	0.9 ± 0.0	0.8 ± 0.0	0.9 ± 0.0	1.0 ± 0.0*
Ovary (%)	—	—	—	—	0.03 ± 0.00	0.03 ± 0.00*	0.02 ± 0.00*	0.03 ± 0.00
Testes (%)	1.0 ± 0.0	1.0 ± 0.0	1.0 ± 0.0	1.1 ± 0.0*	—	—	—	—
Hematology								
Total WBC count (x103/µL)	8.1 ± 0.6	6.9 ± 0.8*	7.3 ± 1.0*	7.8 ± 0.7	7.6 ± 0.7	7.0 ± 0.7	7.1 ± 0.8	7.6 ± 0.8
Clinical chemistry								
AST (IU/L)	66.0 ± 8.8	65.5 ± 8.3	66.3 ± 9.8	70.9 ± 5.9	61.1 ± 4.6	64.8 ± 6.8	64.0 ± 6.2	64.8 ± 1.9
ALT (IU/L)	22.1 ± 5.7	24.1 ± 3.6	23.2 ± 5.1	24.4 ± 5.3	22.1 ± 5.0	24.6 ± 4.6	27.0 ± 7.0	33.0 ± 7.2
BUN (mg/dL)	20.6 ± 2.0	22.2 ± 2.2	22.2 ± 2.2	21.0 ± 3.4	21.5 ± 2.1	23.0 ± 1.5	22.9 ± 1.6	20.9 ± 2.7

Total WBC, Total white blood cell; Alanine aminotransferase - ALT; Aspartate aminotransferase - AST; Blood urea nitrogen - BUN.

Data shown as mean ± SD.

*Statistica significance compared to control, *p* < 0.05.

N = 10 animals/sex/group.

Note: Some values appear to be zero (0.0 or 0.00) due to rounding.

### 3.3 Bacterial reverse mutation assay

All phases of the tests were considered valid. Based on the results of the cytotoxicity tests, concentrations of Organic Reishi M2-102-02 powder or Organic Cordyceps M2-116-04 powder tested in the definitive and independent repeat assays were 50, 100, 500, 1,000, and 5,000 μg/plate, with and without S9. There were no concentration-related or statistically significant treatment-related increases in the number of revertant colonies observed with strains *S. typhimurium* TA1535, TA97a, TA98, and TA100, or *E. coli* WP2 uvrA in both the absence and presence of S9 using the plate incorporation method with either test substance (Table 4, 5, respectively; repeat assays for Organic Reishi M2-102-02 powder and Organic Cordyceps M2-116-04 powder shown in Supplementary Data Sheet S1). Based on the results of this study, Organic Reishi M2-102-02 powder and Organic Cordyceps M2-116-04 powder were negative for the potential to induce gene mutation bacteria with and without metabolic activation.

**TABLE 4:** Results of the bacterial reverse mutation assay with Organic Reishi M2-102-02 powder.

	Revertant colonies per plate									
Concentration (µg/plate)	*Salmonella typhimurium*								*Escherichia coli*	
	TA98		TA100		TA1535		TA97a		WP2 uvrA	
	-S9	+S9 (5%)	-S9	+S9 (5%)	-S9	+S9 (5%)	-S9	+S9 (5%)	-S9	+S9 (5%)
50	28.3 ± 3.3	30.0 ± 3.4	136 ± 3.1	165 ± 3.1	12.8 ± 3.2	10.1 ± 3.3	117 ± 2.1	156 ± 2.8	60.3 ± 2.2	66.1 ± 2.6
100	32.4 ± 3.1	32.0 ± 3.0	138 ± 2.2	129 ± 3.4	14.0 ± 2.3	14.0 ± 3.5	121 ± 2.3	145 ± 3.3	66.5 ± 4.2	65.1 ± 4.3
500	33.8 ± 3.2	35.7 ± 3.0	140 ± 3.2	152 ± 3.5	14.0 ± 2.4	13.6 ± 2.4	121 ± 2.2	135 ± 3.5	66.9 ± 3.0	68.1 ± 3.5
1,000	35.1 ± 4.0	37.0 ± 2.0	146 ± 3.2	155 ± 3.3	14.2 ± 2.1	16.2 ± 2.2	123 ± 2.1	133 ± 2.9	67.1 ± 3.0	70.9 ± 3.2
5,000	38.4 ± 3.2	88.1 ± 3.2	145 ± 3.3	161 ± 3.1	14.7 ± 3.4	16.0 ± 3.0	227 ± 2.2	202 ± 2.7	71.1 ± 3.2	72.0 ± 3.2
Positive control	1705 ± 198*	1724 ± 199*	2,882 ± 140*	2,451 ± 741*	2,161 ± 400*	2,344 ± 390*	1,296 ± 134*	1,276 ± 129*	1,322 ± 123*	1,359 ± 133*
Negative control	32.1 ± 3.1	34.1 ± 3.2	134 ± 18.0	165 ± 16.0	10.8 ± 2.6	151 ± 2.5	128 ± 20.2	149 ± 20.1	73.0 ± 10.1	78.0 ± 1.5

Data shown as mean ± SD.

*Statistical significance compared to control, *p* < 0.05.-S9 positive controls for Strain 98: 2-nitrofluorene (2NF); Strains TA, 97a, TA, 100; TA, 1535: Sodium-azide (NaN3); Strain WP2: methyl-methanesulfonate (MMS).

+S9 (5%) positive controls for Strain 98: Benzo(a)pyrene(BP); Strains TA, 97a, TA, 100; TA, 1535, WP2: 2-aminoanthracene (2AAn).

**TABLE 5:** Results of the bacterial reverse mutation assay with Organic Cordyceps M2-116-04 powder.

	Revertant colonies per plate									
Concentration (µg/plate)	*Salmonella typhimurium*								*Escherichia coli*	
	TA98		TA100		TA1535		TA97a		WP2 uvrA	
	-S9	+S9 (5%)	-S9	+S9 (5%)	-S9	+S9 (5%)	-S9	+S9 (5%)	-S9	+S9 (5%)
50	27.5 ± 5.2	32.3 ± 8.5	138 ± 4.28	164 ± 7.0	12.7 ± 4.5	14.3 ± 4.8	118 ± 2.5	115 ± 3.5	61.2 ± 3.5	60.3 ± 10.0
100	33.4 ± 4.3	36.5 ± 4.8	139 ± 4.6	162 ± 2.1	13.3 ± 3.5	156 ± 4.3	122 ± 4.1	123 ± 4.8	65.5 ± 6.6	61.3 ± 8.5
500	34.5 ± 3.5	35.5 ± 4.7	141 ± 4.5	158 ± 7.5	14.2 ± 3.5	16.4 ± 4.9	122 ± 4.6	127 ± 4.8	67.4 ± 3.3	66.3 ± 7.6
1,000	36.7 ± 4.0	37.8 ± 4.5	145 ± 4.6	163 ± 12.5	14.9 ± 4.5	18.4 ± 5.8	124 ± 4.4	127 ± 4.4	68.3 ± 5.0	67.7 ± 7.6
5,000	37.7 ± 5.5	39.3 ± 5.5	146 ± 5.6	176 ± 9.0	15.3 ± 4.9	18.2 ± 4.7	227 ± 4.2	120 ± 4.2	70.4 ± 5.6	69.7 ± 5.7
Positive control	1706 ± 201*	1,363 ± 201*	2,887 ± 150*	2,867 ± 150*	2,163 ± 403*	2,363 ± 403*	1,298 ± 135*	1,398 ± 135*	1,325 ± 125*	1,425 ± 125*
Negative control	33.4 ± 3.4	33.6 ± 3.4	134 ± 21.0	154 ± 21.0	11.4 ± 2.5	155 ± 2.5	129 ± 23.1	169 ± 23.1	72.3 ± 14.5	777 ± 14.5

Data shown as mean ± SD.

*Statistical significance compared to control, *p* < 0.05.

-S9 positive controls for Strain 98: 2-nitrofluorene (2NF); Strains TA, 97a, TA, 100; TA, 1535: Sodium-azide (NaN3); Strain WP2: methyl-methanesulfonate (MMS).

+S9 (5%) positive controls for Strain 98: Benzo(a)pyrene(BP); Strains TA, 97a, TA, 100; TA, 1535, WP2: 2-aminoanthracene (2AAn).

### 3.4 *In Vivo* mammalian cell micronucleus test

No premature deaths occurred in any male or female mice in any the dose groups administered Organic Reishi M2-102-02 powder or Organic Cordyceps M2-116-04 powder. No clinical signs were observed in the treated animals at any dose level up to 2000 mg/kg bw, including the satellite animals. Treatment with Organic Reishi M2-102-02 powder or Organic Cordyceps M2-116-04 powder did not result in any statistically significant changes in the frequency of micronuclei or micronucleated PCE up to the highest dose tested at 24 and 48 h after administration when compared to the study vehicle control group (Table 6, 7, respectively). The positive control group showed a statistically significant increase in the incidence of micronucleated PCE, confirming the sensitivity and validity of the studies to assess mutagenic activity. Based on the results of this study, Organic Reishi M2-102-02 powder and Organic Cordyceps M2-116-04 powder were negative with respect to the potential for clastogenicity and/or aneugenicity.

**TABLE 6:** Result of the *in vivo* micronucleus test in mice with Organic Reishi M2-102-02 powder.

Treatment group	Dose	Number of PCE with micronuclei per 2000 PCE	PCE/NCE ratio
Vehicle control	10 mL/kg bw, 24 h	0.8 ± 0.6	0.65 ± 0.13
Positive control	50 mg/kg bw, 24 h	51.7 ± 18.0**	0.65 ± 0.13
Organic Reishi M2-102-02 powder	300 mg/kg bw, 24 h	0.8 ± 0.8	0.65 ± 0.13
	1000 mg/kg bw, 24 h	1.1 ± 1.0	0.49 ± 0.31
	2000 mg/kg bw, 24 h	1.2 ± 0.9	0.51 ± 0.14
Organic Reishi M2-102-02 powder satellite	2000 mg/kg bw, 48 h	1.7 ± 1.1	0.52 ± 0.14

Data shown as mean ± SD.

**Statistical significance compared to vehicle control, *p* < 0.01.

PCE: polychromatic erythrocytes; NCE: normochromatic erythrocytes.

Vehicle control–phosphate buffered saline; Positive Control–cyclophosphamide.

**TABLE 7:** Result of the *in vivo* micronucleus test in mice with Organic Cordyceps M2-116-04 powder.

Treatment group	Dose	Number of PCE with micronuclei per 2000 PCE	PCE/NCE ratio
Vehicle control	10 mL/kg bw, 24 h	0.8 ± 0.6	0.65 ± 0.13
Positive control	50 mg/kg bw, 24 h	51.7 ± 18.0**	0.65 ± 0.13
Organic Cordyceps M2-116-04 powder	300 mg/kg bw, 24 h	0.8 ± 0.8	0.65 ± 0.13
	1000 mg/kg bw, 24 h	1.4 ± 0.9	0.49 ± 0.31
	2000 mg/kg bw, 24 h	1.5 ± 0.9	0.51 ± 0.14
Organic Cordyceps M2-116-04 powder satellite	2000 mg/kg bw, 48 h	1.9 ± 1.0	0.52 ± 0.14

Data shown as mean ± SD.

**Statistical significance compared to vehicle control, *p* < 0.01.

PCE: polychromatic erythrocytes; NCE: normochromatic erythrocytes.

Vehicle control–phosphate buffered saline; Positive Control–cyclophosphamide.

## 4 Discussion

This series of studies was conducted to examine the potential acute toxicity, subchronic oral toxicity, and genotoxic potential of Organic Reishi M2-102-02 powder and Organic Cordyceps M2-116-04 powder. In separate studies of the mushroom powders, Organic Reishi M2-102-02 powder and Organic Cordyceps M2-116-04 powder were not acutely toxic, with no effect reported following a single oral dose of 2000 mg/kg bw of either powder. Additionally, Organic Reishi M2-102-02 powder and Organic Cordyceps M2-116-04 powder were not genotoxic in *in vitro* bacterial reverse mutation assays up to 5,000 µg/plate or in *in vivo* mouse micronucleus assays up to 2000 mg/kg bw. Results from the subchronic oral toxicity studies of the Organic Reishi M2-102-02 powder and Organic Cordyceps M2-116-04 powder showed some statistically significant findings; however, for both mushroom powder preparations all changes reported relative to the vehicle control group were attributed to normal and expected variability and were not associated with histopathological findings.

In the subchronic oral toxicity of Organic Reishi M2-102-02 powder, the statistically significant effects included an increase in the absolute spleen weight in female animals in the high dose group without corresponding effects in spleen weight relative to body weight, and thus considered non-adverse (Lewis et al., 2002). Both absolute ovary weights in the mid and high dose groups and ovary relative to body weight in the high dose group were statistically significantly increased relative to control; the increases were <10% different relative to control, thus the effects were determined to be non-treatment related and attributable to normal reproductive cycling resulting in inter-animal variation (Sellers et al., 2007). Testes absolute weight and weight relative to body weight in high dose males were also statistically significantly increased, <10% different than control; the effect was not considered adverse because the magnitude of the change was considered to be within the normal organ weight distribution (World Health Organization, 2015) and no histopathological findings were reported (World Health Organization, 2015; Palazzi et al., 2016; Pandiri et al., 2017). Changes reported in total white blood cell count at all doses compared to the controls, and MCV and MCHC (in the mid and high dose females compared to controls) were considered non-adverse as they were within 20% of control values (World Health Organization, 2015) and the change in AST in high dose males was non-adverse because it was within 50% of the control group value (World Health Organization, 2015).

In the subchronic oral toxicity of Organic Cordyceps M2-116-04 powder, statistically significant findings included changes in ovary and testes absolute weights and weight relative to body weight in female and male rats, respectively, and brain weight relative to body weight in female rats. The effects were <10% different than control thus not considered adverse because the magnitude of the changes were within the normal organ weight distribution (World Health Organization, 2015) and no histopathological findings were reported (World Health Organization, 2015; Palazzi et al., 2016; Pandiri et al., 2017). All other changes did not demonstrate a dose-response and were not considered treatment-related.

The materials that were the subject of testing in these studies were powder preparations of DNA-verified *G. lucidum* mycelial biomass and fruiting body cultured on oats and *C. militaris* mycelial biomass, stroma, and fruiting body cultured on oats produced by M2 Ingredients Inc. Limited information on the potential toxicity of *G. lucidum* and *C. militaris* preparations has been reported in the published literature (Ahmad et al., 2021; Jędrejko et al., 2021). The limited available literature represents various extracts or other preparations of select mushroom components with no standardized main constituents to allow for comparisons across test materials. Materials used in the production of Organic Reishi M2-102-02 powder and Organic Cordyceps M2-116-04 powder meet established product specifications and the fermentation is conducted in a controlled process, thus resulting in production of powders that consistently meet established parameters and reflect only the inherent variability typical of any natural product. However, these powders were developed through fermentation of DNA-verified fungi on organic oats, and thus likely differ in composition from various extracts of wildcraft mushroom and from fungi fermented on a different substrate. Specific cultures and fermentation processes are recognized to affect composition of fermentation preparations (Bidegain et al., 2019); consequently, extracts or powders from different parts of *G. lucidum* and *C. militaris* preparations may vary in toxicity, and data from the available literature may not accurately reflect the composition of the *G. lucidum* and *C. militaris* fermentation preparations on organic oats examined in the studies presented herein. The toxicity studies conducted on the Organic Reishi M2-102-02 and Organic Cordyceps M2-116-04 powders thus provide critical evidence to support the safety of these preparations.

## 5 Conclusion

Acute and repeated dose oral toxicity studies and *in vitro* and *in vivo* genotoxicity assays in rats were conducted to provide toxicological data to support a safety assessment for Organic Reishi M2-102-02 powder and Organic Cordyceps M2-116-04 powder. Organic Reishi M2-102-02 powder and Organic Cordyceps M2-116-04 powder were non-genotoxic and showed no adverse effects up to and including the highest concentration tested in acute and subchronic repeated dose toxicity studies by dietary administration. The 90-day oral toxicity studies support NOAELs for Organic Reishi M2-102-02 powder and Organic Cordyceps M2-116-04 powder of 2000 mg/kg bw/day, the highest dose tested, in male and female rats.

## Data Availability

The original contributions presented in the study are included in the article/Supplementary Material, further inquiries can be directed to the corresponding author.
