# Supplementary material for: A toxicological assessment of Ganoderma lucidum and Cordyceps militaris mushroom powders
Source: Front Toxicol. 2024 Oct 30;6:1469348. doi: 10.3389/ftox.2024.1469348 (PMC11558339; doi:10.3389/ftox.2024.1469348)
Supplement: Supplementary file 1 [file Table1.docx]

**Supplemental Tables**

**Table Summary results of the acute oral toxicity study with Organic Reishi M2-102-02 powder**

| **Dose (mg/kg bw/day)** | | **61** | | | **195** | | | **625** | | | **2000** | | | | |
| --- | --- | --- | --- | --- | --- | --- | --- | --- | --- | --- | --- | --- | --- | --- | --- |
| **Animals No.** | | 1 | 2 | 3 | 1 | 2 | 3 | 1 | 2 | 3 | 1 | 2 | 3 | 4 | 5 |
| **Mortality Observed** | | No | No | No | No | No | No | No | No | No | No | No | No | No | No |
| **Body weight (g)** | **Day 0** | 205 | 198 | 190 | 202 | 199 | 196 | 209 | 196 | 188 | 185 | 192 | 190 | 203 | 205 |
|  | **Day 1** | 203 | 200 | 192 | 205 | 201 | 195 | 209 | 199 | 185 | 188 | 192 | 192 | 204 | 202 |
|  | **Day 7** | 210 | 208 | 189 | 213 | 205 | 199 | 219 | 206 | 195 | 190 | 195 | 194 | 206 | 204 |
|  | **Day 14** | 219 | 204 | 196 | 224 | 211 | 209 | 223 | 201 | 191 | 192 | 196 | 195 | 205 | 206 |
| **Macroscopic Findings (Abnormalities detected)** | | None | None | None | None | None | None | None | None | None | None | None | None | None | None |

**Table Summary of results in the acute oral toxicity study with Organic Cordyceps M2-116-04 powder**

| **Dose (mg/kg bw/day)** | | **61** | | | **195** | | | **625** | | | **2000** | | | | |
| --- | --- | --- | --- | --- | --- | --- | --- | --- | --- | --- | --- | --- | --- | --- | --- |
| **Animals No.** | | 1 | 2 | 3 | 1 | 2 | 3 | 1 | 2 | 3 | 1 | 2 | 3 | 4 | 5 |
| **Mortality Observed** | | No | No | No | No | No | No | No | No | No | No | No | No | No | No |
| **Body weight (g)** | **Day 0** | 205 | 206 | 210 | 200 | 207 | 203 | 211 | 202 | 195 | 198 | 201 | 197 | 199 | 204 |
|  | **Day 1** | 205 | 207 | 210 | 200 | 207 | 203 | 210 | 202 | 196 | 198 | 200 | 198 | 200 | 204 |
|  | **Day 7** | 208 | 210 | 211 | 203 | 210 | 205 | 211 | 205 | 199 | 201 | 202 | 200 | 203 | 206 |
|  | **Day 14** | 210 | 212 | 215 | 205 | 212 | 207 | 214 | 207 | 202 | 204 | 205 | 202 | 205 | 209 |
| **Macroscopic Findings (Abnormalities detected)** | | None | None | None | None | None | None | None | None | None | None | None | None | None | None |

**Table Organ weights in the 90-day toxicity study with Organic Reishi M2-102-02 powder**

|  | Males | | | | Females | | | |
| --- | --- | --- | --- | --- | --- | --- | --- | --- |
| **Dose group (mg/kg bw/day)** | **Control** | **500** | **1000** | **2000** | **Control** | **500** | **1000** | **2000** |
| **Absolute organ weight** | | | | | | | | |
| Adrenals (g) | 0.56 ± 0.03 | 0.61 ± 0.07 | 0.55 ± 0.06 | 0.65 ± 0.01 | 0.54 ± 0.03 | 0.61 ± 0.07 | 0.55 ± 0.06 | 0.64 ± 0.01 |
| Liver (g) | 7.92 ± 0.22 | 8.46 ± 0.05 | 8.89 ± 0.28 | 8.46 ± 0.03 | 7.89 ± 0.30 | 8.48 ± 0.04 | 8.82 ± 0.30 | 8.45 ± 0.03 |
| Kidneys (g) | 1.74 ± 0.04 | 1.87 ± 0.02 | 1.68 ± 0.04 | 1.62 ± 0.06 | 1.72 ± 0.05 | 1.88 ± 0.02 | 1.68 ± 0.04 | 1.60 ± 0.07 |
| Brain (g) | 2.18 ± 0.36 | 2.06 ± 0.19 | 2.07 ± 0.46 | 2.61 ± 0.03 | 2.20 ± 0.31 | 2.09 ± 0.25 | 2.26 ± 0.49 | 2.62 ± 0.03 |
| Heart (g) | 0.85 ± 0.03 | 0.88 ± 0.02 | 0.83 ± 0.03 | 0.98 ± 0.03 | 0.85 ± 0.02 | 0.87 ± 0.02 | 0.82 ± 0.03 | 0.95 ± 0.03 |
| Spleen (g) | 0.81 ± 0.02 | 0.81 ± 0.02 | 0.83 ± 0.02 | 0.83 ± 0.02 | 0.81 ± 0.02 | 0.81 ± 0.02 | 0.82 ± 0.02 | 0.87 ± 0.03* |
| Lungs (g) | 1.78 ± 0.03 | 1.87 ± 0.06 | 1.85 ± 0.04 | 1.87 ± 0.02 | 1.80 ± 0.03 | 1.85 ± 0.06 | 1.85 ± 0.07 | 1.88 ± 0.02 |
| Testes (g) | 2.34 ± 0.04 | 2.46 ± 0.03 | 2.37 ± 0.10 | 2.64 ± 0.04 | - | - | - | - |
| Ovary (mg) | - | - | - | - | 64.7 ± 1.1 | 65.7 ± 3.8 | 65.0 ± 4.8 | 74.0 ± 2.9* |
| **Relative to body weight (%)** | | | | | | | | |
| Adrenals | 0.2% ± 0.0 | 0.2% ± 0.0 | 0.2% ± 0.0 | 0.3% ± 0.0 | 0.2% ± 0.0 | 0.3% ± 0.0 | 0.2% ± 0.0 | 0.3% ± 0.0 |
| Liver | 3.2% ± 0.0 | 3.5% ± 0.0 | 3.6% ± 0.0 | 3.3% ± 0.0 | 3.3% ± 0.0 | 3.6% ± 0.0 | 3.5% ± 0.0 | 3.4% ± 0.0 |
| Kidneys | 0.7% ± 0.0 | 0.8% ± 0.0 | 0.7% ± 0.0 | 0.6% ± 0.0 | 0.7% ± 0.0 | 0.8% ± 0.0 | 0.7% ± 0.0 | 0.6% ± 0.0 |
| Brain | 0.9% ± 0.0 | 0.8% ± 0.0 | 0.8% ± 0.0 | 1.0% ± 0.0 | 0.9% ± 0.0 | 0.9% ± 0.0 | 0.9% ± 0.0 | 1.0% ± 0.0 |
| Heart | 0.3% ± 0.0 | 0.4% ± 0.0 | 0.3% ± 0.0 | 0.4% ± 0.0 | 0.4% ± 0.0 | 0.4% ± 0.0 | 0.3% ± 0.0 | 0.4% ± 0.0 |
| Spleen | 0.3% ± 0.0 | 0.3% ± 0.0 | 0.3% ± 0.0 | 0.3% ± 0.0 | 0.3% ± 0.0 | 0.3% ± 0.0 | 0.3% ± 0.0 | 0.3% ± 0.0 |
| Lungs | 0.7% ± 0.0 | 0.8% ± 0.0 | 0.8% ± 0.0 | 0.7% ± 0.0 | 0.7% ± 0.0 | 0.8% ± 0.0 | 0.7% ± 0.0 | 0.7% ± 0.0 |
| Ovary | - | - | - | - | 0.0% ± 0.0 | 0.0% ± 0.0 | 0.0% ± 0.0 | 0.0% ± 0.0* |
| Testes | 1.0% ± 0.0 | 1.0% ± 0.0* | 1.0% ± 0.0 | 0.0% ± 0.0* | - | - | - | - |

Data shown as mean ± SD weight

* Statistical significance compared to control, p<0.05.

Note: Some values appear to be zero (0.0 or 0.00) due to rounding.

**Table**  **Hematology results in the 90-day repeat dose study with Organic Reishi M2-102-02 powder**

|  | **Dose group (mg/kg bw/day)** | | | | | | | |
| --- | --- | --- | --- | --- | --- | --- | --- | --- |
|  | Males | | | | Females | | | |
| **Parameters** | **Control** | **500** | **1000** | **2000** | **Control** | **500** | **1000** | **2000** |
| Hemoglobin (g/dL) | 14.0 ± 1.2 | 12.7 ± 0.8 | 11.0 ± 0.8 | 12.1 ± 1.1 | 14.6 ± 1.2 | 14.3 ± 0.5 | 11.6 ± 0.7 | 12.5 ± 1.7 |
| Total RBC count (x106/µL) | 7.9 ± 0.4 | 7.9 ± 0.3 | 6.4 ± 0.9 | 6.8 ± 0.9 | 8.0 ± 0.3 | 8.4 ± 0.2 | 6.4 ± 0.7 | 7.4 ± 0.8 |
| Total WBC count (x103/µL) | 6.9 ± 1.2 | 5.0 ± 1.2* | 6.5 ± 0.9 | 6.8 ± 0.7 | 9.5 ± 1.1 | 7.4 ± 1.2* | 7.8 ± 0.9* | 7.7 ± 1.0* |
| PCV (%) | 38.2 ± 0.8 | 43.3 ± 1.1* | 39.4 ± 3.8 | 35.9 ± 3.0 | 42.0 ± 1.5 | 45.0 ± 1.9 | 43.1 ± 2.4 | 43.4 ± 3.8 |
| Platelet count (lakh/cmm) | 670.6 ± 63.7 | 723.8 ± 39.9 | 848.2 ± 87.9* | 690.7 ± 88.6 | 915.3 ± 111.9 | 815.2 ± 67.2 | 866.9 ± 82.4 | 761.5 ± 127.4* |
| MCV (fl) | 41.6 ± 1.5 | 51.6 ± 2.6* | 59.2 ± 1.1* | 56.2 ± 2.2* | 48.0 ± 2.4 | 51.8 ± 1.6 | 60.3 ± 1.1* | 59.7 ± 2.7* |
| MCH (pg) | 16.6 ± 0.5 | 15.7 ± 0.4 | 16.7 ± 1.1 | 18.8 ± 1.2 | 17.7 ± 1.1 | 16.1 ± 0.5 | 17.0 ± 1.1 | 19.6 ± 1.4 |
| MCHC (g/dL) | 28.6 ± 0.7 | 27.9 ± 0.4 | 18.2 ± 1.6* | 31.0 ± 1.0* | 29.0 ± 1.5 | 27.8 ± 0.5 | 33.5 ± 2.6* | 32.3 ± 1.2* |
| Lymphocytes (%) | 62.6 ± 1.3 | 67.1 ± 2.5* | 66.4 ± 4.0* | 62.8 ± 2.5 | 70.1 ± 2.9 | 68.5 ± 2.3 | 70.4 ± 2.0 | 72.6 ± 2.2 |
| Neutrophils (%) | 25.0 ± 2.1 | 28.7 ± 1.3* | 26.6 ± 3.0 | 26.0 ± 2.2 | 29.8 ± 2.8 | 27.7 ± 1.2 | 31.6 ± 3.6 | 32.6 ± 2.9 |
| Monocytes (%) | 1.7 ± 0.4 | 1.5 ± 0.7 | 1.3 ± 0.2 | 0.9 ± 0.1 | 2.5 ± 0.2 | 1.7 ± 0.6 | 1.9 ± 0.3 | 1.5 ± 0.2 |
| Eosinophils (%) | 2.6 ± 0.2 | 2.3 ± 0.4 | 2.6 ± 0.4 | 2.0 ± 0.5 | 3.3 ± 0.3 | 2.1 ± 0.4* | 2.7 ± 0.5 | 2.7 ± 0.7 |

MCH - Mean corpuscular hemoglobin; MCHC - Mean corpuscular hemoglobin concentration; MCV - Mean corpuscular volume; PCV - Packed cell volume; Total RBC - Total red blood cell ; Total WBC - Total white blood cell

Data shown as mean ± SD

* Statistical significance compared to control, p<0.05.

**Table**  **Clinical chemistry results in the 90-day repeat dose study with Organic Reishi M2-102-02 powder**

|  | **Dose group (mg/kg bw/day)** | | | | | | | |
| --- | --- | --- | --- | --- | --- | --- | --- | --- |
|  | Males | | | | Females | | | |
| **Parameters** | **Control** | **500** | **1000** | **2000** | **Control** | **500** | **1000** | **2000** |
| Albumin (g/dL) | 3.4 ± 0.4 | 3.5 ± 0.5 | 3.3 ± 0.7 | 3.1 ± 0.2 | 3.2 ± 0.5 | 3.3 ± 0.3 | 3.6 ± 0.8 | 3.1 ± 0.3 |
| Total Protein (g/dL) | 7.9 ± 0.4 | 8.3 ± 0.3 | 8.4 ± 0.1 | 8.0 ± 0.3 | 7.6 ± 0.7 | 8.1 ± 0.4 | 8.8 ± 0.3 | 7.9 ± 0.6 |
| Bilirubin Total (mg/dL) | 0.3 ± 0.1 | 0.3 ± 0.0 | 0.4 ± 0.1 | 0.3 ± 0.1 | 0.3 ± 0.1 | 0.4 ± 0.1 | 0.3 ± 0.1 | 0.3 ± 0.1 |
| Bilirubin Direct (mg/dL) | 0.3 ± 0.0 | 0.3 ± 0.0 | 0.3 ± 4.3 | 0.3 ± 0.0 | 0.3 ± 0.1 | 0.3 ± 0.0 | 0.3 ± 0.0 | 0.3 ± 0.0 |
| ALT (IU/L) | 22.7 ± 5.3 | 26.9 ± 4.5 | 25.8 ± 13.8 | 33.2 ± 7.5 | 22.9 ± 6.9 | 26.9 ± 4.5 | 28.5 ± 5.9 | 30.2 ± 4.6 |
| ALP (IU/L) | 135.5 ± 15.6 | 136.9 ± 9.8 | 140.6 ± 10.6 | 145.0 ± 0.8 | 136.3 ± 14.0 | 147.1 ± 0.5 | 145.3 ± 0.5 | 134.1 ± 22.0 |
| AST (IU/L) | 57.6 ± 7.0 | 65.6 ± 6.8 | 61.2 ± 2.6 | 69.7 ± 1.6* | 67.4 ± 8.5 | 65.0 ± 10.1 | 57.7 ± 6.3 | 69.8 ± 10.1 |
| BUN (mg/dL) | 21.1 ± 2.8 | 22.3 ± 3.3 | 26.9 ± 0.5* | 20.2 ± 2.5 | 21.7 ± 2.1 | 24.0 ± 1.1 | 28.2 ± 1.8* | 21.4 ± 2.0 |
| Uric acid | 1.1 ± 0.2 | 1.0 ± 0.5 | 1.2 ± 0.2 | 1.0 ± 0.3 | 1.2 ± 0.2 | 1.1 ± 0.5 | 0.9 ± 0.3 | 0.9 ± 0.4 |
| Creatinine (mg/dL) | 0.6 ± 0.0 | 0.7 ± 0.1 | 0.6 ± 2.7 | 0.8 ± 0.1 | 0.6 ± 0.1 | 0.7 ± 0.1 | 0.7 ± 0.1 | 0.6 ± 0.2 |
| Total Cholesterol (mg/dL) | 55.1 ± 2.5 | 54.8 ± 3.0 | 55.2 ± 2.3 | 54.8 ± 1.7 | 54.3 ± 3.4 | 58.2 ± 2.8 | 56.5 ± 4.7 | 53.4 ± 3.0 |
| Glucose (mg/dL) | 155.9 ± 3.2 | 164.8 ± 2.7 | 148.8 ± 5.1 | 160.9 ± 2.2 | 152.3 ± 1.1 | 167.0 ± 11.7 | 150.3 ± 3.0 | 162.1 ± 2.7 |
| Triglycerides | 55.8 ± 4.4 | 58.8 ± 4.1 | 56.3 ± 0.6 | 61.1 ± 4.2 | 58.4 ± 3.8 | 56.6 ± 3.0 | 60.0 ± 4.2 | 66.1 ± 11.3 |
| T3 (nmol/L) | 1.2 ± 0.0 | 1.4 ± 0.0 | 1.5 ± 0.5 | 1.3 ± 0.5 | 1.2 ± 0.0 | 1.3 ± 0.0 | 1.3 ± 0.7 | 1.0 ± 0.4 |
| T4 (nmol/L) | 2.5 ± 0.1 | 2.7 ± 0.1 | 2.4 ± 0.5 | 2.6 ± 0.8 | 2.5 ± 0.0 | 2.7 ± 0.1 | 2.6 ± 0.4 | 2.2 ± 0.7 |
| TSH (ng/mL) | 1.7 ± 0.0 | 1.6 ± 0.1 | 2.2 ± 0.5 | 1.6 ± 0.5 | 1.7 ± 0.1 | 1.6 ± 0.1 | 2.1 ± 0.5 | 1.5 ± 0.5 |

Alanine Aminotransferase (ALT); Alkaline phosphatase (ALP); Aspartate aminotransferase (AST); Blood urea nitrogen (BUN); Thyroid stimulating hormone (TSH)

Data shown as mean ± SD

* Statistical significance compared to control, p<0.05

Note: Some values appear to be zero (0.0) due to rounding.

**Table Organ weights in the 90-day toxicity study with Organic Cordyceps M2-116-04 powder**

|  | Males | | | | Females | | | |
| --- | --- | --- | --- | --- | --- | --- | --- | --- |
| **Dose group (mg/kg bw/day)** | **Control** | **500** | **1000** | **2000** | **Control** | **500** | **1000** | **2000** |
| **Absolute organ weight** | | | | | | | | |
| Adrenals (g) | 0.58 ± 0.03 | 0.61 ± 0.00 | 0.59 ± 0.04 | 0.55 ± 0.02 | 0.60 ± 0.02 | 0.61 ± 0.00 | 0.61 ± 0.00 | 0.63 ± 0.04 |
| Liver (g) | 7.86 ± 0.28 | 8.47 ± 0.01 | 8.39 ± 0.06 | 7.90 ± 0.06 | 7.95 ± 0.16 | 8.47 ± 0.01 | 8.46 ± 0.01 | 7.81 ± 0.15 |
| Kidneys (g) | 1.70 ± 0.05 | 1.88 ± 0.08 | 1.87 ± 0.14 | 1.85 ± 0.08 | 1.73 ± 0.10 | 1.88 ± 0.12 | 1.85 ± 0.11 | 1.80 ± 0.15 |
| Brain (g) | 2.11 ± 0.06 | 2.05 ± 0.05 | 2.07 ± 0.05 | 2.23 ± 0.12 | 2.12 ± 0.03 | 2.03 ± 0.04 | 2.13 ± 0.03 | 2.40 ± 0.11* |
| Heart (g) | 0.86 ± 0.00 | 0.82 ± 0.01 | 0.78 ± 0.03 | 1.01 ± 0.02 | 0.86 ± 0.05 | 0.82 ± 0.01 | 0.78 ± 0.02 | 1.01 ± 0.01 |
| Spleen (g) | 0.81 ± 0.01 | 0.81 ± 0.04 | 0.78 ± 0.04 | 0.81 ± 0.03 | 0.81 ± 0.01 | 0.83 ± 0.01 | 0.80 ± 0.02 | 0.83 ± 0.01 |
| Lungs (g) | 1.91 ± 0.01 | 1.81 ± 0.17 | 1.79 ± 0.05 | 1.93 ± 0.01 | 1.93 ± 0.02 | 1.89 ± 0.04 | 1.80 ± 0.12 | 1.90 ± 0.03* |
| Testes (g) | 2.57 ± 0.04 | 2.50 ± 0.01* | 2.30 ± 0.06 | 2.49 ± 0.06* | - | - | - | - |
| Ovary (mg) | - | - | - | - | 66.0 ± 4.9 | 61.0 ± 3.0* | 59.9 ± 0.0* | 69.9 ± 0.0* |
| **Relative to body weight (%)** | | | | | | | | |
| Adrenals | 0.2% ± 0.0 | 0.2% ± 0.0 | 0.2% ± 0.0 | 0.2% ± 0.0 | 0.2% ± 0.0 | 0.3% ± 0.0 | 0.2% ± 0.0 | 0.3% ± 0.0 |
| Liver | 3.2% ± 0.0 | 3.4% ± 0.0 | 3.4% ± 0.0 | 3.2% ± 0.0 | 3.3% ± 0.0 | 3.5% ± 0.0 | 3.4% ± 0.0 | 3.2% ± 0.0 |
| Kidneys | 0.7% ± 0.0 | 0.8% ± 0.0 | 0.8% ± 0.0 | 0.7% ± 0.0 | 0.7% ± 0.0 | 0.8% ± 0.0 | 0.7% ± 0.0 | 0.7% ± 0.0 |
| Brain | 0.9% ± 0.0 | 0.8% ± 0.0 | 0.8% ± 0.0 | 0.9% ± 0.0 | 0.9% ± 0.0 | 0.8% ± 0.0 | 0.9% ± 0.0 | 1.0% ± 0.0* |
| Heart | 0.3% ± 0.0 | 0.3% ± 0.0 | 0.3% ± 0.0 | 0.4% ± 0.0 | 0.4% ± 0.0 | 0.3% ± 0.0 | 0.3% ± 0.0 | 0.4% ± 0.0 |
| Spleen | 0.3% ± 0.0 | 0.3% ± 0.0 | 0.3% ± 0.0 | 0.3% ± 0.0 | 0.3% ± 0.0 | 0.3% ± 0.0 | 0.3% ± 0.0 | 0.3% ± 0.0 |
| Lungs | 0.8% ± 0.0 | 0.7% ± 0.0 | 0.7% ± 0.0 | 0.8% ± 0.0 | 0.8% ± 0.0 | 0.8% ± 0.0 | 0.7% ± 0.0 | 0.8% ± 0.0 |
| Ovary | - | - | - | - | 0.03% ± 0.00 | 0.03% ± 0.00* | 0.02% ± 0.00* | 0.03% ± 0.00 |
| Testes | 1.0% ± 0.0 | 1.0% ± 0.0 | 1.0% ± 0.0 | 1.1% ± 0.0* | - | - | - | - |

Data shown as mean ± SD weight

* Statistical significance compared to control, p<0.05.

Note: Some values appear to be zero (0.0 or 0.00) due to rounding.

**Table**  **Hematology results in the 90-day repeat dose study with Organic Cordyceps M2-116-04 powder**

|  | **Dose group (mg/kg bw/day)** | | | | | | | |
| --- | --- | --- | --- | --- | --- | --- | --- | --- |
|  | Males | | | | Females | | | |
| **Parameters** | **Control** | **500** | **1000** | **2000** | **Control** | **500** | **1000** | **2000** |
| Hemoglobin (g/dL) | 13.1 ± 1.1 | 12.6 ± 0.7 | 12.4 ± 1.4 | 12.3 ± 0.7 | 13.1 ± 1.3 | 12.6 ± 0.7 | 12.7 ± 0.7 | 12.3 ± 0.7 |
| Total RBC count (x106/µL) | 7.7 ± 0.4 | 6.9 ± 0.4 | 8.1 ± 0.5 | 6.6 ± 0.4 | 7.9 ± 0.3 | 7.1 ± 0.2 | 7.0 ± 0.5 | 6.7 ± 0.2 |
| Total WBC count (x103/µL) | 8.1 ± 0.6 | 6.9 ± 0.8* | 7.3 ± 1.0* | 7.8 ± 0.7 | 7.6 ± 0.7 | 7.0 ± 0.7 | 7.1 ± 0.8 | 7.6 ± 0.8 |
| PCV (%) | 46.6 ± 1.6 | 43.9 ± 1.1 | 43.9 ± 1.1 | 41.9 ± 0.9 | 45.8 ± 2.7 | 42.7 ± 0.9 | 42.7 ± 1.1 | 40.6 ± 1.0 |
| Platelet count (lakh/cmm) | 708.0 ± 109.3 | 836.7 ± 27.7 | 895.2 ± 52.1 | 790.2 ± 56.9 | 795.3 ± 159.2 | 793.6 ± 56.9 | 813.6 ± 65.7 | 808.2 ± 27.7 |
| MCV (fl) | 59.3 ± 2.9 | 54.0 ± 1.4 | 61.6 ± 2.5 | 56.3 ± 1.5 | 58.2 ± 3.6 | 54.9 ± 1.5 | 58.8 ± 1.7 | 58.1 ± 1.5 |
| MCH (pg) | 15.7 ± 0.7 | 16.7 ± 0.5 | 16.7 ± 0.5 | 17.0 ± 0.5 | 15.7 ± 0.7 | 16.0 ± 0.6 | 16.3 ± 0.6 | 16.2 ± 0.6 |
| MCHC (g/dL) | 27.1 ± 0.6 | 27.0 ± 1.0 | 27.1 ± 1.1 | 29.6 ± 1.0 | 27.3 ± 0.7 | 27.6 ± 0.8 | 27.0 ± 0.8 | 28.8 ± 0.8 |
| Lymphocytes (%) | 67.8 ± 1.6 | 66.5 ± 1.2 | 68.0 ± 1.2 | 69.6 ± 1.2 | 67.3 ± 2.4 | 66.9 ± 1.6 | 67.9 ± 1.6 | 68.5 ± 1.6 |
| Neutrophils (%) | 28.1 ± 2.4 | 28.6 ± 2.2 | 32.0 ± 3.3 | 29.6 ± 2.2 | 31.8 ± 1.6 | 28.6 ± 3.3 | 28.2 ± 2.3 | 27.7 ± 3.3 |
| Monocytes (%) | 2.2 ± 0.5 | 2.1 ± 0.3 | 1.9 ± 0.3 | 2.1 ± 0.3 | 1.8 ± 0.5 | 1.9 ± 0.3 | 1.7 ± 0.3 | 1.8 ± 0.3 |
| Eosinophils (%) | 3.0 ± 0.5 | 2.6 ± 0.2 | 2.6 ± 0.2 | 3.1 ± 0.6 | 2.7 ± 0.7 | 2.6 ± 0.6 | 2.2 ± 0.6 | 3.0 ± 0.2 |

MCH - Mean corpuscular hemoglobin; MCHC - Mean corpuscular hemoglobin concentration; MCV - Mean corpuscular volume; PCV - Packed cell volume; Total RBC - Total red blood cell ; Total WBC - Total white blood cell

Data shown as mean ± SD

* Statistical significance compared to control, p<0.05.

Note: Some values appear to be zero (0.0) due to rounding.

**Table**  **Clinical chemistry results in the 90-day repeat dose study with Organic Cordyceps M2-116-04 powder**

|  | **Dose group (mg/kg bw/day)** | | | | | | | |
| --- | --- | --- | --- | --- | --- | --- | --- | --- |
|  | Males | | | | Females | | | |
| **Parameters** | **Control** | **500** | **1000** | **2000** | **Control** | **500** | **1000** | **2000** |
| Albumin (g/dL) | 3.2 ± 0.4 | 3.5 ± 0.4 | 3.4 ± 0.5 | 3.3 ± 0.4 | 3.0 ± 0.1 | 3.7 ± 0.6 | 3.4 ± 0.7 | 3.2 ± 0.3 |
| Total Protein (g/dL) | 7.3 ± 0.4 | 7.3 ± 0.4 | 7.4 ± 0.7 | 7.6 ± 0.5 | 7.6 ± 0.2 | 7.7 ± 0.2 | 7.7 ± 0.6 | 7.6 ± 0.3 |
| Bilirubin Total (mg/dL) | 0.3 ± 0.1 | 0.3 ± 0.1 | 0.3 ± 0.1 | 0.4 ± 0.1 | 0.3 ± 0.0 | 0.3 ± 0.1 | 0.3 ± 0.1 | 0.3 ± 0.1 |
| Bilirubin Direct (mg/dL) | 0.2 ± 0.0 | 0.2 ± 0.1 | 0.2 ± 0.1 | 0.2 ± 0.0 | 0.2 ± 0.0 | 0.2 ± 0.0 | 0.2 ± 0.0 | 0.2 ± 0.0 |
| ALT (IU/L) | 22.1 ± 5.7 | 24.1 ± 3.6 | 23.2 ± 5.1 | 24.4 ± 5.3 | 22.1 ± 5.0 | 24.6 ± 4.6 | 27.0 ± 7.0 | 33.0 ± 7.2 |
| ALP (IU/L) | 143.0 ± 0.5 | 143.0 ± 0.8 | 142.6 ± 1.3 | 143.3 ± 0.9 | 143.0 ± 0.5 | 143.3 ± 0.5 | 142.5 ± 1.0 | 143.3 ± 0.7 |
| AST (IU/L) | 66.0 ± 8.8 | 65.5 ± 8.3 | 66.3 ± 9.8 | 70.9 ± 5.9 | 61.1 ± 4.6 | 64.8 ± 6.8 | 64.0 ± 6.2 | 64.8 ± 1.9 |
| BUN (mg/dL) | 20.6 ± 2.0 | 22.2 ± 2.2 | 22.2 ± 2.2 | 21.0 ± 3.4 | 21.5 ± 2.1 | 23.0 ± 1.5 | 22.9 ± 1.6 | 20.9 ± 2.7 |
| Uric acid | 1.1 ± 0.5 | 1.2 ± 0.5 | 1.2 ± 0.5 | 1.0 ± 0.3 | 1.0 ± 0.4 | 0.9 ± 0.3 | 0.9 ± 0.3 | 0.8 ± 0.3 |
| Creatinine (mg/dL) | 0.5 ± 0.1 | 0.6 ± 0.0 | 0.6 ± 0.1 | 0.6 ± 0.1 | 0.6 ± 0.0 | 0.6 ± 0.1 | 0.6 ± 0.1 | 0.7 ± 0.1 |
| Total Cholesterol (mg/dL) | 54.7 ± 2.9 | 55.8 ± 2.8 | 55.4 ± 2.7 | 55.8 ± 1.8 | 55.8 ± 2.6 | 55.0 ± 3.8 | 55.5 ± 4.4 | 54.4 ± 4.9 |
| Glucose (mg/dL) | 156.0 ± 1.3 | 155.9 ± 2.7 | 155.9 ± 2.7 | 154.5 ± 1.8 | 156.2 ± 1.6 | 154.0 ± 2.5 | 154.0 ± 2.5 | 156.2 ± 1.5 |
| Triglycerides | 58.3 ± 3.8 | 58.0 ± 3.0 | 57.4 ± 2.0 | 57.5 ± 6.1 | 57.0 ± 4.1 | 57.4 ± 5.0 | 56.5 ± 3.4 | 61.8 ± 6.4 |
| T3 (nmol/L) | 1.6 ± 0.6 | 2.0 ± 0.5 | 2.0 ± 0.5 | 2.0 ± 0.5 | 1.7 ± 0.6 | 2.3 ± 0.3 | 2.4 ± 0.3 | 2.3 ± 0.3 |
| T4 (nmol/L) | 5.8 ± 1.6 | 7.2 ± 1.5 | 6.7 ± 1.4 | 7.1 ± 1.5 | 6.8 ± 1.6 | 6.1 ± 1.7 | 5.4 ± 1.6 | 6.8 ± 1.3 |
| TSH (ng/mL) | 4.4 ± 1.7 | 5.4 ± 0.7 | 5.7 ± 2.6 | 5.9 ± 2.1 | 4.9 ± 0.9 | 3.7 ± 1.4 | 6.6 ± 2.3 | 4.8 ± 1.5 |

Alanine Aminotransferase (ALT); Alkaline phosphatase (ALP); Aspartate aminotransferase (AST); Blood urea nitrogen (BUN); Thyroid stimulating hormone (TSH)

Data shown as mean ± SD

Note: Some values appear to be zero (0.0) due to rounding.
